# Herbal Agents versus Ethylene Diamine Tetra Acetic Acid on Removal of the Smear Layer—A Systematic Review of In Vitro Studies

**DOI:** 10.3390/ijerph19116870

**Published:** 2022-06-03

**Authors:** Kavalipurapu Venkata Teja, Krishnamachari Janani, Abdullah Ali Alqahtani, Ali Robaian, Feras Alhalabi, Khalid A. Merdad, Mohammad Khursheed Alam, Deepti Shrivastava, Jerry Jose, Kumar Chandan Srivastava

**Affiliations:** 1 Department of Conservative Dentistry and Endodontics, Mamata Institute of Dental Sciences, Bachupally, Hyderabad 500090, Telangana, India; metejaendo@gmail.com; 2 Department of Conservative Dentistry and Endodontics, SRM Institute of Science and Technology, SRM Dental College, Chennai 600089, Tamil Nadu, India; 3 Department of Conservative Dental Sciences, College of Dentistry, Prince Sattam Bin Abdulaziz University, Alkharj 11942, Saudi Arabia; aa.alqahtani@psau.edu.sa (A.A.A.); ali.alqahtani@psau.edu.sa (A.R.); f.alhalabi@psau.edu.sa (F.A.); 4 Endodontic Department, Faculty of Dentistry, King Abdulaziz University, Jeddah 80200, Saudi Arabia; kmerdad@kau.edu.sa; 5 Department of Preventive Dentistry, College of Dentistry, Jouf University, Sakaka 72345, Saudi Arabia; mkalam@ju.edu.sa; 6 Center for Transdisciplinary Research (CFTR), Saveetha Dental College, Saveetha Institute of Medical and Technical Sciences, Saveetha University, Chennai 600077, Tamil Nadu, India; 7 Department of Public Health, Faculty of Allied Health Sciences, Daffodil lnternational University, Dhaka 1205, Bangladesh; 8 Private Practice, Ernakulam District, Aluva 683106, Kerala, India; jerryjosekavungal@gmail.com; 9 Oral Medicine and Radiology, Department of Oral and Maxillofacial Surgery and Diagnostic Sciences, College of Dentistry, Jouf University, Sakaka 72345, Saudi Arabia

**Keywords:** endodontics, disinfection, EDTA, herbal agents irrigants, smear layer, root canal treatment, natural components

## Abstract

This systematic review aimed to compare the efficacy of herbal agents with ethylene diamine tetraacetic acid (EDTA) in removing the smear layer during root canal instrumentation. The research question in the present study was to assess: “Is there a significant difference in reducing smear layer comparing EDTA and herbal agents?” Electronic databases (PubMed, Scopus, and Web of Science) were searched from their start dates to April 2022 using strict inclusion and exclusion criteria, and reviewed following PRISMA (Preferred Reporting Items for Systematic Reviews and Meta-Analyses) 2020 guidelines. Only in vitro studies comparing herbal agents with EDTA were included in the current systematic review. Two reviewers independently assessed the included articles. A total of 625 articles were obtained from an electronic database. Eighteen papers were included for review of the full text, out of which, ten papers were excluded because they did not meet the inclusion criteria. Finally, eight articles were included in the systematic review. The present systematic review considered only in vitro studies; hence, the result cannot be completely translated to strict clinical conditions. The results of the present systematic review have shown that *quixabeira, morindacitrifolia, oregano* extract, and neem show better smear layer removal compared to other herbal agents, whereas they showed reduced smear layer removal when compared with EDTA. Although, it was seen that most of the included studies did not report a high quality of evidence. Hence, the present systematic review concludes that herbal agents have reported to show inferior smear layer removal when compared to EDTA. Thus, as far as herbal based alternatives are concerned, there is no highest level of evidence to state its real benefit when used as a chelating root canal irrigant.

## 1. Introduction

In endodontics, there is an enormous amount of literature on the use of conventional irrigants as root canal disinfectants. To target the complete elimination of microorganisms from the root canal, importance should be focused on the efficiency of chemo-mechanical disinfection. Eventually, during canal preparation, inadvertently, smear layer formation is bound to occur. The smear layer is considered an amorphous substance that consists of inorganic dentin, odontoblastic process, and the necrotic and viable pulp [1]. The smear layer can be of two types: it can either be forced on the superficial dentin or plugged into the dentinal tubules. The thickness of the superficial smear layer can be up to 2–5 μm [2,3,4]. Previous reports have shown that the smear layer tends to plug into dentinal tubules due to adhesive and capillary action, which hinders the efficiency of the irrigant and the sealing ability.

Moreover, these conventional antimicrobial agents, such as sodium hypochlorite, cannot remove the inorganic dentin layer from the root canal system. To overcome this, the use of chelating agents becomes mandatory, and for this purpose, various chelating agents, such as ethylene diamine tetra acetic acid (EDTA), maleic acid, and citric acid, have been employed [5,6]. EDTA is considered a conventional irrigant, as it is most commonly employed to eliminate the smear layer, but over the recent decade, there is literature stating that maleic acid possesses better clinical efficiency than EDTA [6]. Apart from chelating agents, studies have also shown that sodium hypochlorite can remove the smear layer [7].

To date, there has been no systematic review to assess the efficiency of smear layer removal of herbal agents in comparison with EDTA and sodium hypochlorite. Therefore, this systematic review was undertaken to investigate the effect of herbal irrigants compared to routinely used irrigants. This systematic review intends to address the research question on the efficiency of smear layer removal of herbal agents with conventional agents (EDTA and sodium hypochlorite).

## 2. Materials and Methods

This systematic review was conducted following the PRISMA 2020 (Preferred Reporting Items for Systematic Reviews and Meta-Analyses) guidelines. PICOS was based on: population: human extracted teeth infected with E. Faecalis; intervention: experimental studies using herbal root canal irrigants for smear layer removal; comparison: experimental studies using conventional root canal irrigant NaoCl, EDTA for smear layer removal; outcome: removal of smear layer efficiency; study type: in vitro study.

### 2.1. Inclusion Criteria and Exclusion Criteria

The included in vitro studies were performed on extracted teeth. Herbal agents in comparison with NaoCl, EDTA for smear layer removal were considered inclusion criteria, and animal studies, review articles, case reports, and case series were excluded from this current systematic review.

### 2.2. Search Strategy

A detailed search strategy was performed for the identification of included studies. The combination of vocabulary and free text search was performed in a PubMed search up to April 2022. Search terms related to root canal dentin; endodontic treatment; irrigants; herbal agents; smear layer removal; and NaOCl, EDTA were used to search for potential articles. Other databases used for the search were from Scopus and the Cochrane Library. Full-text articles in the English language were only applied for the initial phase of the article search (Table 1).

### 2.3. Selection of Studies

Based on exclusion and inclusion criteria, the studies were included with the aid of a software manager (Rayyan Systems Inc., Cambridge, MA 02142, USA), and the data were reviewed independently by two reviewers (K.J., K.V.T.), and in case of disagreement, a third reviewer (J.J.) sorted the consensus.

### 2.4. Data Extraction

Data extraction was performed by (K.J., K.V.T.), and the eligibility of the studies was assessed following full-text articles. Articles were included that compared conventional smear layer removal agents (EDTA, NaOCL) with herbal agents. Variables, such as teeth selection, positive and negative control, canal preparation size, irrigation protocol, choice of irrigants, volume and concentration of irrigant, choice of the needle, irrigant activation, method of evaluation, magnification, and scoring criteria, were assessed.

### 2.5. Risk of Bias

Risk of bias was performed to assess the specific issues about the review’s potential bias. The risk of bias was scored as “low” when the details of the parameters mentioned above were mentioned with no ambiguity, but when there was ambiguity, they were scored as unclear. When no details were mentioned, it was scored as “high”.

## 3. Results

**Figure 1:**
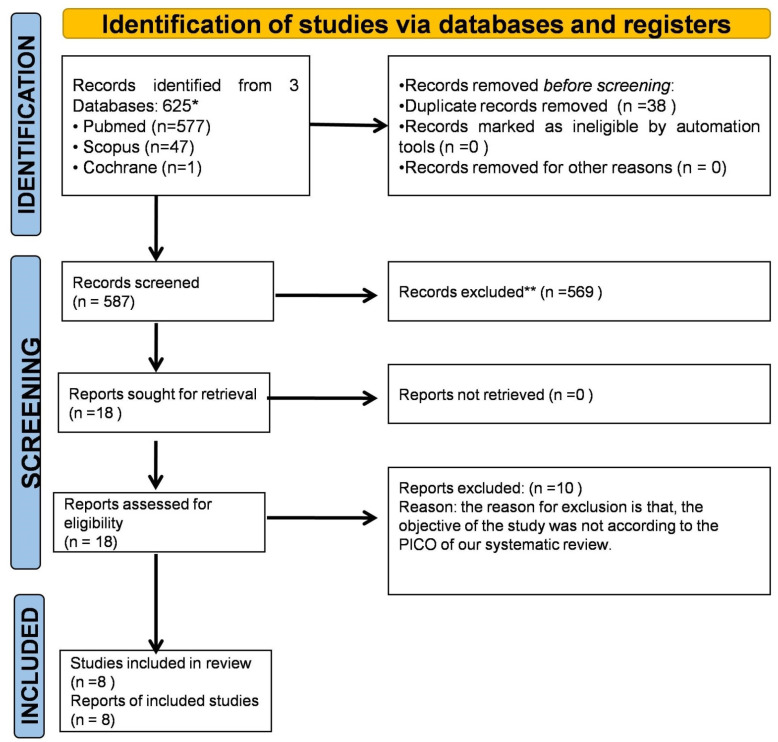
PRISMA 2020 flow diagram for new systematic reviews which included searches of databases and registers only. * Consider, if feasible to do so, reporting the number of records identified from each database or register searched (rather than the total number across all databases/registers). ** If automation tools were used, indicate how many records were excluded by a human, and how many were excluded by automation tools.

The search resulted in 625 papers from PubMed, Scopus, and Web of Science. Duplicates were removed, resulting in 587 papers. A total of 569 papers were excluded because they were out of scope. Eighteen papers were included for review of the full text. Hand searching and reference linkage did not result in any additional papers. Ten papers were excluded because they did not meet the inclusion criteria. Eight articles were included for further analysis to inform this review (Table 2). A summary of the article selection is presented as a flowchart, based on PRISMA guidelines (Figure 1). General characteristics of the included articles were tabulated for eight studies on smear layer removal (Table 3).

### 3.1. Assessment of Smear Layer Removal

A. Size of apical preparation

Three studies used the hand-filing method with a minimum of 30 k file apical preparation of the canal [8,10,14]. The remaining studies used a rotary system for canal preparation [9,12,13,15]. Apical preparation size was prepared up to 25 0.06 in one study [15], 50 0.06 in one study [13], and 35 0.06 in two studies [9,12] (Table 4).

B. Irrigation protocol

No standard irrigation protocol was performed about removing the smear layer amongst all the included studies.

C. Volume and time of irrigation

One study did not mention the volume of root canal irrigant used [9], and three studies did not mention the time of irrigation [9,10,11]. Detailed information regarding the volume and time of irrigation is provided in Table 4.

D. Choice of irrigation needle and irrigant activation

Out of the included studies, five studies did not mention the gauge of the needle used for the disinfection of the root canal [8,9,10,11,13]. Only three studies mentioned the gauge of the needle [12,14,15]. Kumar et al. [14] used a 25-gauge needle, Susan et al. [15] used 28-gauge, and a 30-gauge needle was used in Chhabra et al. [12] amongst all the studies, and only two studies mentioned the vent of the needle used [12,15]. When considering the irrigant activation, none of the studies used irrigant activation devices for smear layer removal, apart from Chhabra et al. [12] (Table 4).

E. Method of smear layer assessment

All the included studies performed Scanning Electron Microscopy (S.E.M.) analysis to assess smear layer removal. Five studies reported the smear layer assessment at all three levels of the root canal system [8,9,11,12,15]. Two studies did not mention the levels of assessment [13,14] (Table 4).

F. Magnification level and scoring criteria

Two studies did not mention the level of magnification at which the smear layer was assessed [12,14]. Two studies did not mention the scoring criteria they used for smear layer removal assessment [11,13]. One study used Romes et al. criteria [14], whereas the other two studies used Hulsmann et al. criteria [8,12]. The results of this present systematic review for smear layer removal efficiency have been tabulated (Table 5).

Discussion of results of the included studies, among the studies that mentioned the level assessment, five studies performed the evaluation at all three levels, namely coronal, middle, and apical [8,9,11,12,15], whereas Candeiro et al. [10] evaluated only middle and apical. Three studies evaluated at 2000× [8,9,15] and two studies at 1000× magnification [10,12]. Only one study used a magnification of 8000× [13]. Out of the eight included studies, five studies reported herbal agents as inferior to conventional agents [8,12,13,14,15] (Table 4).

### 3.2. Risk of Bias

The Joanna Briggs Institute (J.B.I.) criteria for risk of bias was modified according to in vitro studies by evaluating the domains of the present review, such as experimental condition (control groups, sampling methods), evidence on ethical approval, incomplete data (needle size, design, and time and volume of irrigation), blinding, standardization, and reporting of data. Blinding in these studies implies blinding of the evaluator.

**Figure 2:**
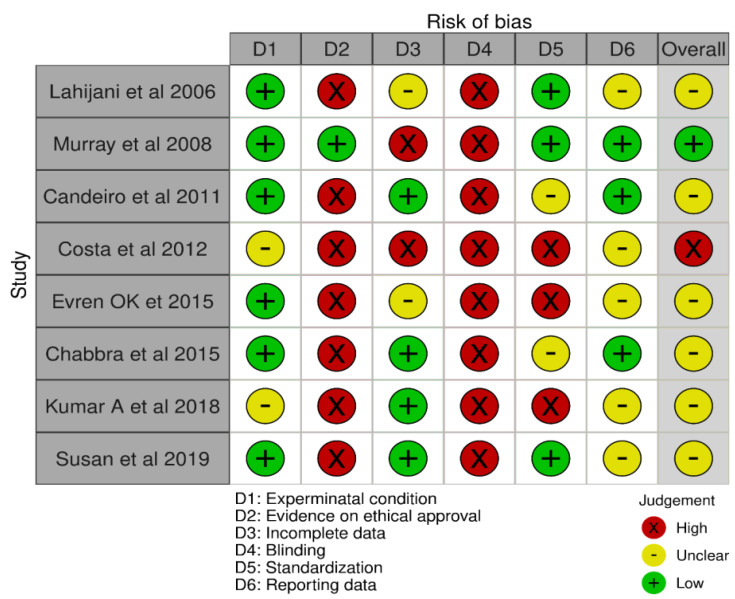
Risk of bias.

Figure 2 depicts the risk of the bias plot. On reviewing the smear layer removal studies, the authors opine that a few essential parameters need to be addressed. Parameters, such as smear layer assessment at different levels in the sample, the range of magnification for the evaluation, standardized protocol, volume of the irrigant, and choice of the needle, were not disclosed in the studies. None of the studies obtained ethical committee approval, apart from Murray et al. [9]. Only lahijani et al. [8] reported blinding in their study, whereas this was missing in the other included studies (Table 6).

## 4. Discussion

The success of endodontic treatment depends on the three-dimensional seal of the root canal system. To achieve this, optimal canal preparation, the use of disinfectant with antimicrobial efficacy and pulp-dissolving ability, along with the use of chelating agents are needed. The smear layer formed following mechanical canal preparation can plug into the dentinal tubules to a depth ranging from 40 to 110 μm [1]. Hence, it is of utmost importance to remove this smear layer to allow optimal infiltration of the irrigant to the intricacies of the root canal system, facilitating the adequate penetration of intracanal medicament.

There are still two schools of thought on removing or maintaining the smear layer. Some reports proposed that maintaining the smear layer intact prevents the further ingress of microorganisms [16], but the authors of this systematic review opine that complete removal of the smear layer is mandatory to attain the three-dimensional seal. This can be achieved by maximum disinfection with the appropriate use of chelators, such that it aids in the penetration of the sealer into the dentin, and enhanced adhesion to the obturating material [17]. Failure to attain this three-dimensional seal may lead to microleakage, eventually leading to endodontic treatment failure.

Over the years, the widely used agents for the disinfection of the root canal include sodium hypochlorite as the primary root canal irrigant, and EDTA as a chelating agent. Sodium hypochlorite is a non-specific proteolytic agent possessing antibacterial properties, and dissolves the remnant pulp tissue [18]. Apart from this, it causes the dissolution of organic components of dentin. It has been already proved in the endodontic literature three decades ago that the combination of sodium hypochlorite and EDTA enhanced the removal of smear layer [19]. When EDTA is combined with sodium hypochlorite, the resulting solution’s activity spreads throughout the rest of the pulp tissues, along with antibacterial properties [20]. A study by landolo et al. reported that heating the hypochlorite to 180 °C was sufficient enough to provide a clean canal without the use of EDTA [21]

The thickness of the smear layer depends on the type of instrument used for preparation. Compared to rotary cutting instruments, hand instruments produced less smear layer formation [22]. When rotary and the reciprocating cutting instruments are compared, studies have shown rotary instruments to form a minimal smear layer [23]. This is because rotary instruments’ configuration is designed to be strenuous to attain contact of the instrument in the long oval and non-rounded canals, which eventually leads to reduced smear layer formation in rotary instruments [24]. The depth and packing density of the smear layer also varies greatly depending on whether the dentin is cut dry or wet, the amount and composition of the irrigating solution used, and the kind and speed of the instrument employed, according to a study by Pashley in 1984 [25]

As we know that the use of EDTA plays a significant role in the removal of the smear layer from the root canal system, it was also reported to tend to induce dentinal erosion, and increase the chance of perforation during instrumentation depending on the concentration volume and contact time [26]. Several studies have shown that EDTA usage for a contact time of 1 min was required to remove the smear layer [27,28,29]. Among the included article in this systematic review, Kumar et al. [9] used the contact time of 45 s, and also, the volume of the irrigant used differed, ranging from 3 mL to 8 mL.

The choice of the needle gauge and design of the needle vent do influence the irrigant to reach the intricacies of the root canal system [30]. In order to achieve this, it is mandatory to use no less than a 28- or 30-gauge needle with a side vent to avoid inadvertent extrusion of the irrigant. In some instances, conventional syringe needle irrigation fails to reach the third apical intricacy of the root canal system, which warrants the use of irrigation activation to increase the flow and distribution. Activation of the irrigant causes acoustic streaming and cavitation, enabling the irrigant to reach the inaccessible areas of the canal [31,32,33]. All the included studies used S.E.M. for analysis at different levels, namely coronal, middle, and apical third. All included studies showed that the remanence of the smear layer is present in the apical part, which is attributed to confounding factors. Studies showed that liquid EDTA promotes enhanced smear layer removal due to reduced surface tension [34]. Moreover, the addition of surfactant decreases the surface tension, and enhances the wettability [35,36].

Over the last two years, research has focused on the use of preheated chelating agents in their efficiency in the removal of the smear layer, and in improving the bond strength [37]. Better results were shown when preheated EDTA was used [37]. The above-mentioned was in corroboration with another study which had mentioned similar results [38]. It has been reported in the literature that preheating the irrigating solutions lowers the surface tension of the dentinal tubules, allowing for improved penetration into the tubules and efficient smear layer removal [39,40]. The authors of this review make a standpoint that more clinical trials are required to uncover the symbiotic effects of preheated hypochlorite and EDTA. Moreover, all the details of the study need to be mentioned and focused on keeping the risk of bias low. Furthermore, the authors of this review believe that the experiments were performed with standardized protocol, but might not have reported the intricate details since these are in vitro studies.

### 4.1. Quantitative Review

Assessing the smear layer removal, 6% Morinda showed a similar result to 6% NaoCl with 17% EDTA [11]. Quixbeira and Neam [15] also showed a similar result as compared with conventional irrigating agents. The authors of this review opine contradictory results in the included studies; there could possibly be a difference in the volume of the irrigant used and the contact time in those studies. The smear layer removal efficiency of oregano extract was similar to that of the conventional agent [12]. The other agents, such as chamomile and tulsi, showed lesser efficiency than the comparison group [8,9].

Hence, it is impossible to conclude that one herbal agent is a better alternative irrigant for smear layer removal, as there are variables in the included studies.

### 4.2. Qualitative Review

Meta-analysis could not be performed due to the heterogeneity of the included articles and variation in the included studies.

### 4.3. Inference

Regarding smear layer removal efficiency, herbal agents (tea tree oil, tulsi, chamomile) showed lesser efficiency than EDTA. *Quixabeira, MorindaCitrifolia, oregano* extract, and neem showed better smear layer removal. The authors of this review infer that the herbal agents cannot be a substitute to conventional agents, as the included studies report incomplete data and a lack of standardization.

### 4.4. Limitation and Future Inference

The present systematic review was at the in vitro level of analysis; therefore, the result cannot translate the exact clinical conditions. Studies should concentrate on the irrigant activation, concentration, type, volume, and contact time of these herbal agents, and concentrate on the precipitate formation when used with conventional irrigating solutions.

## 5. Conclusions

This systematic review concludes that despite quixabeira, morindacitrifolia, oregano extract, and neem showing better smear layer removal compared to other herbal agents, reduced smear layer removal was evident when compared with EDTA. Hence, the present systematic review concludes that herbal agents have reported to show inferior smear layer removal when compared to EDTA.

## Figures and Tables

**Table 1:** Search strategy.

PubMed	(((extracted teeth) AND (root canal treatment [MeSH Terms])) OR (endodontic treatment [MeSH Terms])) AND (herbal irrigants [MeSH Terms])) OR (herbal root canal irrigants)) OR (EDTA [MeSH Terms])) OR (Ethylenediaminetetraaceticacid [MeSH Terms])) OR (sodium hypochlorite solution [MeSH Terms])) OR (sodium hypochlorite root canal irrigant)) AND (smear layer removal)
Scopus	(TITLE-ABS-KEY (root canal therapy) OR TITLE-ABS-KEY (endodontic treatment) OR TITLE-ABS-KEY (root canal irrigants) AND TITLE-ABS KEY (sodium hypochlorite irrigant) OR ALL (ethylenediaminetetraaceticacid) OR ALL (edta) AND TITLE-ABS-KEY (smear layer removal))
Cochrane library	#1 Endodontic treatment #2 Root canal treatment #3 Root canal irrigant #4 Sodium Hypochlorite irrigant #5 EDTA #6 Herbal irrigants #7 Smear layer removal

**Table 2:** List of included articles.

Author	Title of Included Article
Lahijani et al. 2006 [8]	The effect of German chamomile (*Marticariarecutita* L.) extract and tea tree (*Melaleucaalternifolia* L.) oil used as irrigants on removal of smear layer: a scanning electron microscopy study
Murray et al. 2008 [9]	Evaluation of Morinda Citrifolia as an Endodontic Irrigant
Candeiro et al. 2011 [10]	A comparative scanning electron microscopy evaluation of smear layer removal with apple cidar vinegar and sodium hypochlorite associated with EDTA
Costa et al. 2012 [11]	In vitro evaluation of the root canal cleaning ability of plant extracts and their antimicrobial action
Chabbra et al. 2015 [12]	Smear layer removal efficacy of combination of herbal extracts in two different ratios either alone or supplemented with sonic agitation: An in vitro scanning electron microscope study
Evren OK et al. 2015 [13]	Antibacterial and smear layer removal capability of oregano extract solution
Kumar A et al. 2018 [14]	Comparative Evaluation of Antibacterial and Smear Layer Removal Efficacy of Two Different Herbal Irrigants: An in vitro Study
Susan et al. 2019 [15]	Intra radicular Smear Removal Efficacy of Triphala as a Final Rinse Solution in Curved Canals: A Scanning Electron Microscope Study

**Table 3:** Smear layer assessment.

Author and Year	Selection of Teeth	Sample Size	Herbal Irrigant	Positive Control	Negative Control	Other Irrigant
Lahijani et al. 2006 [8]	Single rooted permanent teeth	N = 40	Group C: hydroalcoholic extract of German chamomile group D: tea tree oil	Group B: 2.5% NaoCl with 17% EDTA	Group A: sterile distilled water	Group E: 2.5% NaoCl alone
Murray et al. 2008 [9]	Permanent Single Rooted Premolar	N = 60	Group 1: 6% MorindaCitrifolia Juice (MCJ) with a flush of 17% EDTA, followed by a final flush of MCJ Group 2: 6% MCJ mixed equally with 2% CHX with a flush of EDTA and final flush of MCJ/2% CHX Group 3: 6% MCJ with a flush of saline, followed by a final flush of MCJ	Group 4: 6% NaoCl with a flush of 17% EDTA, followed by a final flush of 6% NaoCl	Group 6: Sterile Saline	Group 5: 2% Chlorhexidine
Candeiro et al. 2011 [10]	Maxillary and mandibular molars	N = 40	Group A—Apple vinegar Group B—Apple vinegar and 17% EDTA as final rinse	Group C—1% NaOCl and 17%EDTA as a final rinse	Group D: Saline	-
Costa et al. 2012 [11]	Single rooted permanent teeth	N = 20	Group 1: 50% Aroeira-Da-Praia Group 2: 50% Quixabeira	Group 3: 2.5% NaoCl with 17% EDTA	Not mentioned	-
Evren OK et al. 2015 [13]	Permanent maxillary central incisors	N = 180	Group 9: 1% OES + distilled water Group 10: 2% OES + distilled water Group 11: 5% OES + distilled water Group 12: 1% OES + 17% EDTA + distilled water Group 13: 2% OES + 17% EDTA + distilled water Group 14: 5% OES + 17% EDTA + distilled water	Group 8: 5.25% NaoCl + 17% EDTA + distilled water	Group 15: sterile saline + 17% EDTA + distilled water	-
Chabbra et al. 2015 [12]	Single canal teeth	N = 50	Group C—Combination of *Citrus aurantifolia* and *Sapindusmukorossi* in 1:1 ratio Group D—Combination of *Citrus aurantifolia* and *Sapindusmukorossi* in 1:1 ratio supplemented with sonic agitation Group E—Combination of *Citrus aurantifolia* and *Sapindusmukorossi* in 2:1 ratio Group F—Combination of *Citrus aurantifolia* and *Sapindusmukorossi* in 2:1 ratio supplemented with sonic agitation	Group B—17% ethylenediaminetetraacetic acid	Group A— Distilled water	
Kumar A et al. 2018 [14]	Maxillary central incisors	N = 120 Antimicrobial efficacy (n = 60), smear layer removal efficacy (n = 60).	Group IIB: 25% Neem extract (n = 20) Group IIC: 25% Tulsi extract (n = 20)	Group IIA: 17% EDTA (n = 20)	Not mentioned	-
Susan et al. 2019 [15]	Mandibular first molar	N = 74	Group 3: Triphala premixed Group 4: Triphala premixed(Sonic activation) Group 5: Triphala premixed (Ultrasonic activation) Group 6: 3% Triphala in 10% DMSO Group 7: 5% Triphala in 10% DMSO Group 8: 10% Triphala in 10% DMSO Group 9: 10% citric acid Group 10:10% DMSO	Group 2: 17% EDTA	Group 1: normal saline	-

**Table 4:** Methodology assessment for smear layer.

Author and Year	Root Canal Preparation (Instruments Used and Size of Preparation)	Irrigation Protocol	Volume of Irrigant	Time of Irrigation	Needle Used for Irrigation	Irrigant Activation Devices Used
Lahijani et al. 2006 [8]	K file up to 30 apical preparation	No protocol was mentioned In group A, C, D, and E, the intra instrumentation irrigant was the same as the final flush irrigant In group B, 2.5% Naocl was followed by 17% EDTA as a final flush	Intra instrumentation— 2 mL Final flush-10 mL	Intra instrumentation 10 s Final flush— 2 min	Not mentioned in the study	Nil
Murray et al. 2008 [9]	Protaper up to 35.06	No protocol was mentioned Group 1: 6% Morinda Citrifolia Juice (MCJ) with a flush of 17% EDTA, followed by a final flush of MCJ Group 2: 6% MCJ mixed equally with 2% CHX with a flush of EDTA and final flush of MCJ/2% CHX Group 3: 6% MCJ with a flush of saline, followed by a final flush of MCJ Group 4: 6% NaoCl with a flush of 17% EDTA, followed by a final flush of 6% NaoCl	Not mentioned in the study	Not mentioned in the study	Not mentioned in the study	Nil
Candeiro et al. 2011 [10]	Up to 45 K file	No protocol was mentioned	2ml of irrigating solution at every change of file	Not mentioned	Not mentioned	Not mentioned
Costa et al. 2012 [11]	Preparation size was not mentioned	No protocol was mentioned Group 1 and Group 2—respective irrigant was used. Group 3—2.5% sodium hypochlorite, followed by 17% EDTA, and then, 3% saline solution.	Group 1 and 2—3 mL	Not mentioned in the study	Not mentioned in the study	Nil
Evren OK et al. 2015 [13]	Protaper up to 50 0.6	No protocol mentioned Following the canal preparation, irrigation was performed with the respective irrigant	**Group 8** 3 mL 5.25% Naocl 3 mL 17% EDTA 5 mL distilled water **Group 9** 3 mL 1% OES 5 mL distilled water **Group 10** 3 mL 2% OES 5 mL distilled water **Group 11** 3 mL 5% OES 5 mL distilled water **Group 12** 3 mL 1% OES 3 mL 17% EDTA 5 mL distilled water **Group 13** 3 mL 2% OES 3 mL 17% EDTA 5 mL distilled water **Group 14** 3 mL 5% OES 3 mL 17% EDTA 5 mL distilled water **Group 15** 3 mL sterile saline 3 mL 17% EDTA 5 mL distilled water	1min 1 min 1min 1 min 1 min 1 min 1 min 1 min 1 min 1 min 1 min 1 min 1 min 1 min 1 min 1 min 1 min 1 min 1 min 1 min 1 min	Not mentioned	Nil
Chabbra et al. 2015 [12]	Apical size 35, 0.06 taper using nickel titanium files	No protocol was mentioned	During instrumentation, each root canal irrigated using 2 mL and final rinse 3 mL of solution corresponding to its group	5 min	30 gauge Side vented	Sonic activation performed in group D and F
Kumar et al. 2018 [14]	K file up to 30 size apical preparation	No protocol mentioned (following the canal preparation, irrigation was performed with respective irrigant)	6ml	45 s	25-gauge needle	Nil
Susan et al. 2019 [15]	Up to apical size 25, 0.06 taper using rotary nickel–titanium files	1 mL of the irrigant was used for canal irrigation after each instrument No protocol was mentioned	8 mL during biomechanical preparation, and 5 mL for final rinse	3 min	28-gauge side vented needle	Not mentioned

**Table 5:** Result assessment on smear layer.

Author and Year	Smear Layer Evaluation Method	Assessment of Level of Root Canal	Magnification	Scoring Criteria	Statistical Analysis	% of Open Dentinal Tubules	Outcome
Lahijani et al. 2006 [8]	SEM analysis	Cervical, middle, and apical level of canal	2000× and 5000×	Hulsmann et al. criteria	Kruskal–Wallis and Mann–Whitney U tests	2.5% Naocl with 17%EDTA—no smear layer detected 2.5% Naocl—moderate smear layer, mainly in apical third Chamomile—moderate to heavy smear layer in apical third. Moderate to thin in middle and coronal sections	2.5% Naocl followed by 17% EDTA showed better results in smear layer when compared to chamomile extract. The least effective was tea tree oil
Murray et al. 2008 [9]	SEM analysis	Cervical, middle, and apical level of canal	2000×	Modified semi-quantitative visual criterion by Madison and Hokett criteria	X^2^ statistical test	% of complete smear layer removal 6% Naocl with 17% EDTA final flush—80% smear removed (middle and coronal area) MCJ with 17% EDTA final flush—70% smear removed (middle and coronal area) MCJ with saline—20, 30, 20% smear removed (apical, middle, coronal, respectively)	6% MCJ was equally effective as 6% Naocl when 17% EDTA was used as final flush.
Candeiro et al. 2011 [10]	SEM analysis	Middle and apical third	×1000	Vale et al. criteria	Kruskal–Wallis and Dunn’s test Wilcoxon test	Middle third less smear layer removal than apical third	Apple cider vinegar with EDTA showed better smear layer removal, followed by apple cider vinegar and NaoCl/EDTA
Costa et al. 2012 [11]	SEM analysis	Cervical, middle, and apical level of canal	Not mentioned	Not mentioned	Kruskal–Wallis analysis	Naocl with EDTA—more accumulation of smear layer in apical third than middle and coronal Aroeira-Da-Praia—more accumulation of smear layer in apical third and middle than coronal Quixabeira—less accumulation of smear layer than Naocl with EDTA	Quixabeira was found to be more effective in apical smear layer removal than Naocl with EDTA
Evren OK et al. 2015 [13]	SEM analysis	Levels not mentioned	×8000	Not mentioned	Kruskal–Wallis and Mann–Whitney U tests	1 or 2 or 5% oregano extract solution followed by 17% EDTA showed maximum removal of smear layer, whereas 1 or 2 or 5% oregano extract alone failed to remove the smear layer	5.25% NaoCl followed by 17%EDTA showed similar effect on smear layer removal as that of 1 or 2 or 5% oregano extract solution
Chabbra et al. 2015 [12]	SEM analysis	Coronal, middle, and apical third	×1000	Hulsmann et al. criteria	One-way analysis of variance. Tukey’s post hoc test	Mean score of smear layer 17% EDTA (Group B) Coronal = 1.4 Middle = 2.2 Apical = 1.8 Group F (*Citrus aurantifolia* and *Sapindusmukorossi* in 2:1 ratio with sonic agitation) Coronal = 1.6 Middle = 2.6 Apical = 2.1	17% EDTA and Combination of *Citrus aurantifolia* and *Sapindusmukorossi* in 2:1 ratio with sonic agitation showed maximum removal of smear layer
Kumar A et al. 2018 [14]	SEM analysis	Levels not mentioned	Not mentioned	Rome et al. criteria	One-way analysis of variance. Tukey’s post hoc test	Mean of smear layer EDTA = 1.20 Neem leaf extract = 1.90 Tulsi extract = 2.70	EDTA showed maximum removal of smear layer followed by neem extract. The least was observed with tulsi.
Susan et al. 2019 [15]	SEM analysis	Coronal, middle, and apical third	×2000	Caron et al.	Not mentioned	Mean score of smear layer Group 5: Triphala premixed (Ultrasonic activation) = 1.6 ± 0.63	Triphala showed least amounts of smear in all thirds of the root canal, with mean values of 1.6 ± 0.63, similar to that of EDTA

**Table 6:** Risk of bias assessment on smear layer (predetermined criteria based on JBI criteria).

Author and Year	Experimental Condition (Control Groups, Sampling Methods)	Evidence on Ethical Approval	Incomplete Data (Needle Size, Design, Time & Volume of Irrigation)	Blinding	Standardization	Reporting Data
Lahijani et al. 2006 [8]	Low	High	Unclear (type of needle and needle design not mentioned)	High (not mentioned)	Low	Unclear (percentage or mean value of remaining smear layer adherent not mentioned)
Murray et al. 2008 [9]	Low	Low	High (volume of irrigant, time of irrigation, and type of needle used not mentioned)	High (not mentioned)	Low	Low
Candeiro et al. 2011 [10]	Low	High	High (time and needle gauge and design not mentioned)	High	Unclear (irrigation protocol not mentioned)	Low
Costa et al. 2012 [11]	Unclear (negative control not mentioned)	High	High (volume of irrigant, type of needle used not mentioned)	High (not mentioned)	High (range of magnification for SEM analysis not mentioned)	Unclear (percentage or mean value of remaining smear layer adherent not mentioned)
Evren OK et al. 2015 [13]	Low	High	Low	High	Unclear (irrigation protocol not mentioned)	Low
Chabbra et al. 2015 [12]	Low	High	Unclear (gauge and needle design not mentioned)	High (not mentioned)	High (smear layer assessment at different levels and criteria for assessment, type of needle used not mentioned)	Unclear (percentage or mean value of remaining smear layer adherent not mentioned)
Kumar A et al. 2018 [14]	Unclear (negative control not mentioned)	High	Low	High (not mentioned)	High (irrigation protocol was not mentioned, smear layer assessment at different levels, and range of magnification not mentioned)	Unclear (percentage or mean value of remaining smear layer adherent not mentioned)
Susan et al. 2019 [15]	Low	High	Low	High	Low	Unclear (statistical test not reported)

## Data Availability

Not applicable.
